# Innate Immunity Never “NODs” Off: NLRs Regulate the Host Anti‐Viral Immune Response

**DOI:** 10.1111/imr.13429

**Published:** 2025-01-29

**Authors:** Mackenzie K. Woolls, Madeline D. Mott, Cassandra S. Poole, Julia A. Gregory, Hannah M. Ivester, Irving Coy Allen

**Affiliations:** ^1^ Graduate Program in Translational Biology, Medicine, and Health Virginia Tech Roanoke Virginia USA; ^2^ Biomedical and Veterinary Sciences Graduate Program, Virginia Maryland College of Veterinary Medicine Virginia Tech Blacksburg Virginia USA; ^3^ Department of Biological Sciences Undergraduate Program, College of Biological Sciences Virginia Tech Blacksburg Virginia USA; ^4^ Department of Basic Science Education Virginia Tech Carilion School of Medicine Roanoke Virginia USA; ^5^ The Virginia Tech Center for Emerging, Zoonotic and Arthropod‐Borne Pathogens Virginia Tech Blacksburg Virginia USA

**Keywords:** antiviral immunity, inflammasome, inflammation, NOD‐like receptors, virus

## Abstract

A robust innate immune response is essential in combating viral pathogens. However, it is equally critical to quell overzealous immune signaling to limit collateral damage and enable inflammation resolution. Pattern recognition receptors are critical regulators of these processes. The cytosolic nucleotide‐binding domain leucine‐rich repeat (NLR; NOD‐like receptor) family of pattern recognition receptors plays essential roles in the sensing of viral pathogen‐associated molecular patterns and is best characterized for itsr pro‐inflammatory biological functions. Specifically, these include the formation of multi‐protein complexes, defined as inflammasomes or NODosomes that regulate the production of IL‐1beta, IL‐18, and pyroptosis, or the induction of NF‐ΚB signaling. While these biological effects are inherently pro‐inflammatory, it is also important to recognize that other NLR family members conversely function to negatively regulate inflammation through modulating signaling initiated by other families of pattern recognition receptors. Mechanistically, these unique NLRs also form multiprotein complexes that act to attenuate a variety of biological signaling pathways, such as the inhibition of NF‐ΚB. This inhibition facilitates inflammation resolution and functions to restore cellular homeostasis. Despite extensive characterization of individual NLR family members, the mechanisms of immune system regulation are highly nuanced and remain enigmatic. This is especially true for non‐inflammasome‐forming, regulatory NLRs. Here, we discuss recent findings associated with NLR family members that play essential roles in the host immune response to viruses and mechanisms by which these pattern recognition receptors may function to regulate antiviral immunity.

## 1 Introduction

**FIGURE 1:**
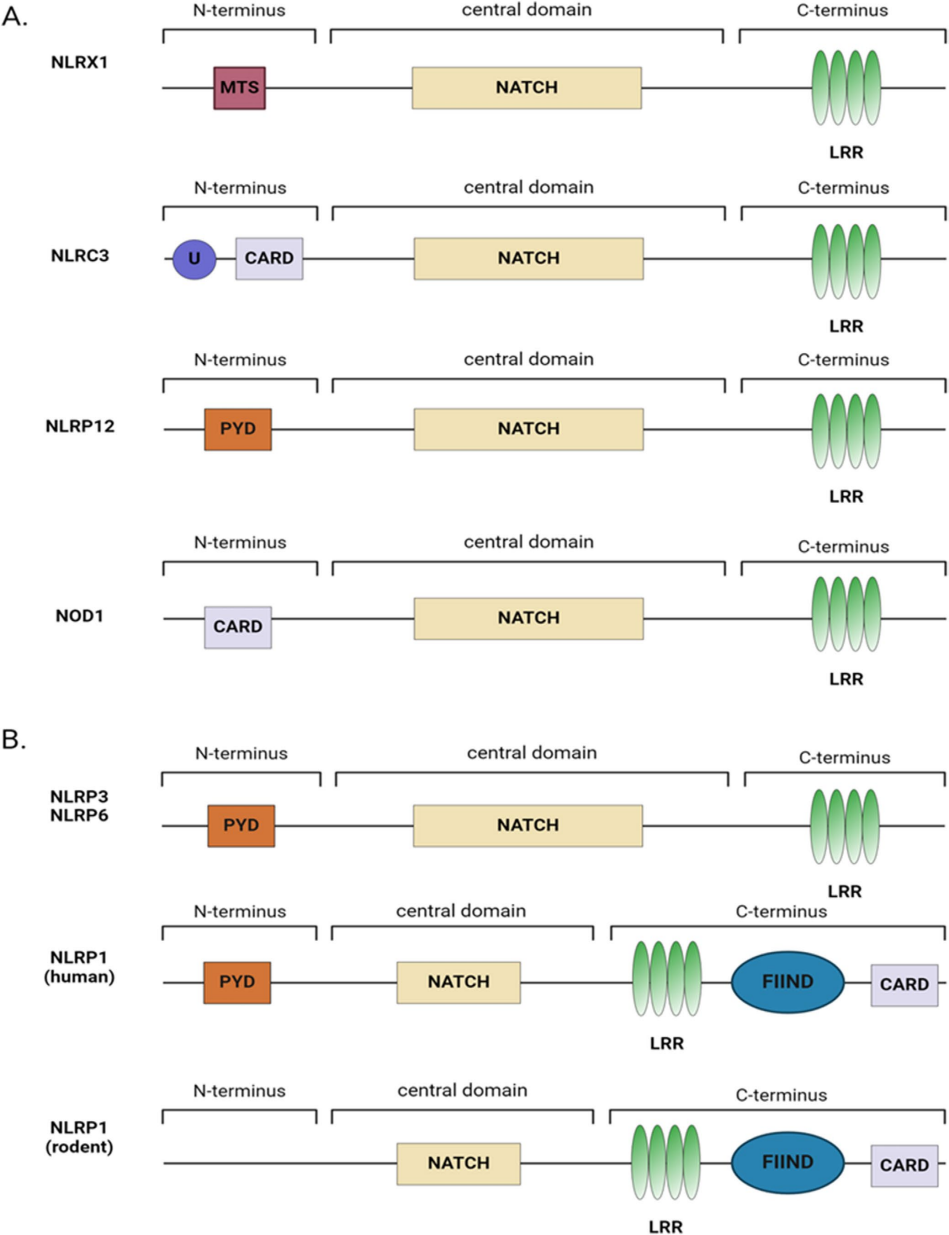
Structure of regulatory and inflammasome‐forming NLRs. (A) Structure of example regulatory NLRs: NLRX1, NLRC3, NLRP12, NOD1. (B) Structure of example inflammasome‐forming NLRs: NLRP3, NLRP6, and NLRP1 (human and rodent). The domains noted are as follows: CARD, Caspase activation and recruitment domain; FIIND, Function‐to‐find domain; LRR, Leucine‐rich repeats; MTS, Mitochondria‐targeting sequence; PYD, Pyrin effector domain; U, Undetermined structure.

Pattern recognition receptors sense broad pathogen‐associated molecular patterns (PAMPs) associated with viral infection. Pathogen recognition initiates the biochemical signaling pathways that drive the ensuing innate immune response and also enhances adaptive immune system activation. The pattern recognition receptors are either membrane bound (i.e., Toll‐like receptors (TLRs)) or cytosolic (i.e., RIG‐I‐like Helicase Receptors (RLRs), C‐Type lectin receptors (CLR) and NOD‐like Receptors (NLRs)). In addition to these defined pattern recognition receptors, there are several unique cytoplasmic receptors that have also been described that do not fit within a currently characterized family (i.e., cyclic GMP‐AMP synthase (cGAS); stimulator of IFN genes (STING), and DNA‐dependent activator of IFN‐regulatory factors (DAI)) [1]. These can be broadly grouped as xLRs. The contributions of TLRs, RLRs, and many of the xLRs are relatively well studied in the context of host‐virus interactions. However, the roles of many of the NLRs are less defined. In humans, the NLR family is composed of 22 distinct members, whereas mice have 34 NLRs [2]. NLRs are characterized by their tripartite domain structure that is composed of a variable but limited repertoire of N‐terminal domains, a central nucleotide‐binding (NACHT) domain, and a C‐terminal domain composed of leucine‐rich repeats (Figure 1) [3]. Ligand sensing occurs through the LRR domain, which enables a conformation change and the subsequent interaction with adaptor and/or effector proteins that facilitate downstream intracellular signaling. All of the NLRs thus far associated with pathogen recognition serve as scaffolds for the formation of multi‐protein signaling complexes.

NLRs can be divided into three subgroups based on their best‐characterized biological functions. The most heavily studied subgroup includes NLRs that form an inflammasome with the adaptor molecule ASC and are associated with the cleavage and processing of IL‐1β, IL‐18, and pyroptosis. There are at least 8 well‐characterized NLRs in this subgroup, including NLRP1, NLRP3, NLRP6, NLRP12, and NLRC4, some of which are discussed in more detail below. The second subgroup is reproductive NLRs that function during embryogenesis, including NLRP7, NLRP14, NLRP4, NLRP5, and NLRP11. Reproductive NLRs are generally less characterized due to embryonic lethality and/or a lack of orthologs in mice. While inflammasome formation is not the primary mechanism of biological action for most of these family members, some can form inflammasomes under limited circumstances following virus exposure. The third NLR subgroup is defined as regulatory NLRs. Regulatory NLRs do not form inflammasomes. Rather, regulatory NLRs form unique multi‐protein complexes, such as the “NODosome”, or regulate the activities of other pattern recognition receptors. NODosomes can include not only other pattern recognition receptors, such as TLRs, RLRs, and xLRs, but also other NLR family members. The regulatory activities of these sensors can be pro‐inflammatory. These positive regulators of inflammation include NOD1 and NOD2. Conversely, the regulatory functions can attenuate immune signaling to control excessive or overzealous inflammation. These negative regulatory NLRs include NLRC3, NLRP12, and NLRX1 [4, 5, 6, 7, 8]. Each of these regulatory NLRs modulates diverse signaling pathways, including NF‐κB and interferon signaling, through mechanisms that are highly relevant following virus infection.

While the NLR field has rapidly advanced over the last decade, many of the NLRs remain only superficially characterized, and deep mechanistic insight is lacking. This is true of approximately half of the NLR family. Without deep mechanistic insight, it is difficult to define clinical relevance and therapeutic potential. This is especially true for the members of the reproductive and regulatory NLR subgroups. This review will focus on our current knowledge related to the role of specific NLR family members in the host immune response to viruses and will present emerging concepts associated with their biological functions modulating viral immune signaling.

## 2 Inflammasome Forming NLRS

**FIGURE 2:**
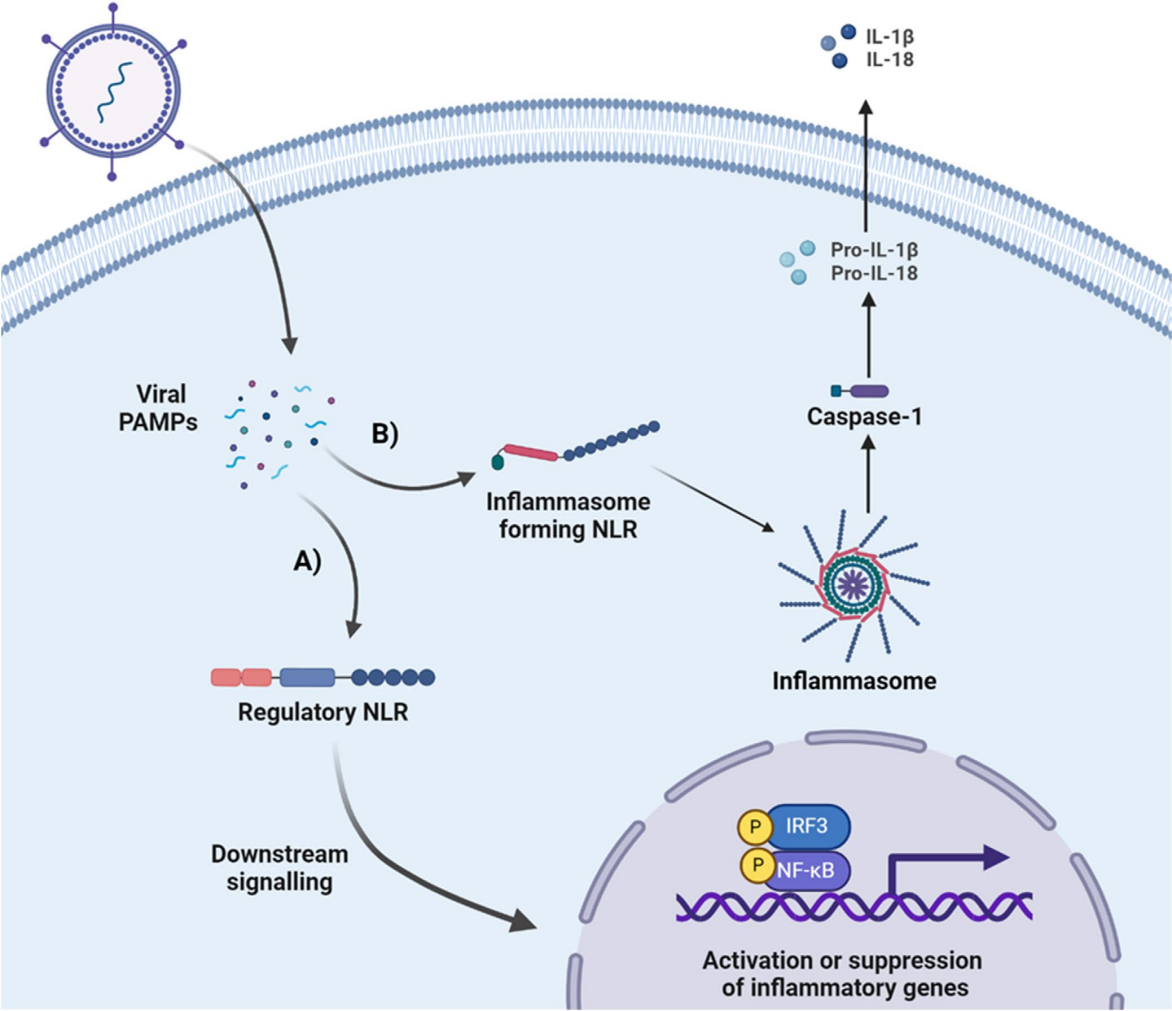
Viral activation of regulatory and inflammasome‐forming NLR pathways. Following viral entry, pathogen‐associated molecular patterns, including viral nucleic acids and glycoproteins, are either directly or indirectly sensed by NLRs. (A) Regulatory NLRs form multiprotein complexes with various proteins in the cytosol to either activate or inhibit inflammatory signaling pathways, such as NF‐κB and interferon. (B) Binding of PAMPs to inflammasome‐forming NLRs results in the formation of the multi‐protein inflammasome complex, which activates caspase‐1 and the subsequent cleavage of IL‐1β and IL‐18.

The best‐characterized subgroup of NLRs is those that form so‐called “inflammasomes” (Figure 2). Inflammasome‐forming NLRs include NLRP3, NLRP1, NLRP6, NLRC4, and a range of additional NLRs that are lesser characterized. Inflammasome formation is driven by recognition of pathogen‐associated molecular patterns (PAMPs) and damage‐associated molecular patterns (DAMPs). Sensor activation leads to the oligomerization of the adaptor protein PYCARD (also known as ASC), which includes a pyrin domain (PYD) and a caspase recruitment domain (CARD). These domains complex with the reciprocal domains on the NLR protein through either PYD‐PYD or CARD‐CARD interactions. Once complexed with ASC, pro‐caspases are then recruited, including pro‐caspase‐1, which is the best characterized. This multi‐protein complex, consisting of NLRs, ASC, and pro‐Caspase‐1, forms the functional inflammasome structure. Following inflammasome formation, pro‐caspase‐1 is cleaved to become active in the cell cytosol, ultimately resulting in the cleavage of the caspase‐1 target proteins, pro‐IL‐1β and pro‐IL‐18. Cleavage by caspase‐1 is required for the activation of both of these cytokines. Complementing the activation of IL‐1β and IL‐18, caspase‐1 has also been shown to cleave Gasdermin‐D (GMSD) between the N‐terminus and carboxyterminal C domain, resulting in the N‐terminus activation and initiation of a specific type of inflammatory cell death termed pyroptosis [9].

Inflammasome activation and pyroptosis are critical biological mechanisms that mediate the host immune response following virus exposure. The cytokine IL‐1β is a driver of the fever response, controls immune cell recruitment and activation, induces an interferon‐like state at the site of infection, and can restrict the formation of syncytia between cells to restrict virus transmission. The cytokine IL‐18 also controls immune cell recruitment and activation, as well as regulates epithelial cell barrier regeneration and repair, augments natural killer cell‐mediated cytotoxicity, and maintains the T helper 1‐driven microenvironment at the site of virus infection. Together, these cytokines ensure a robust and optimal pro‐inflammatory innate immune response. However, it is also important to note that the uncontrolled activation and release of these cytokines can result in significant collateral damage, resulting in loss of tissue function. Thus, a delicate balance must be maintained to eradicate the viral pathogen and then resolve the ensuing innate immune response. Of the inflammasome‐forming NLRs, four have been defined and characterized in the context of viral infection.

### 2.1 NLRP3

**FIGURE 3:**
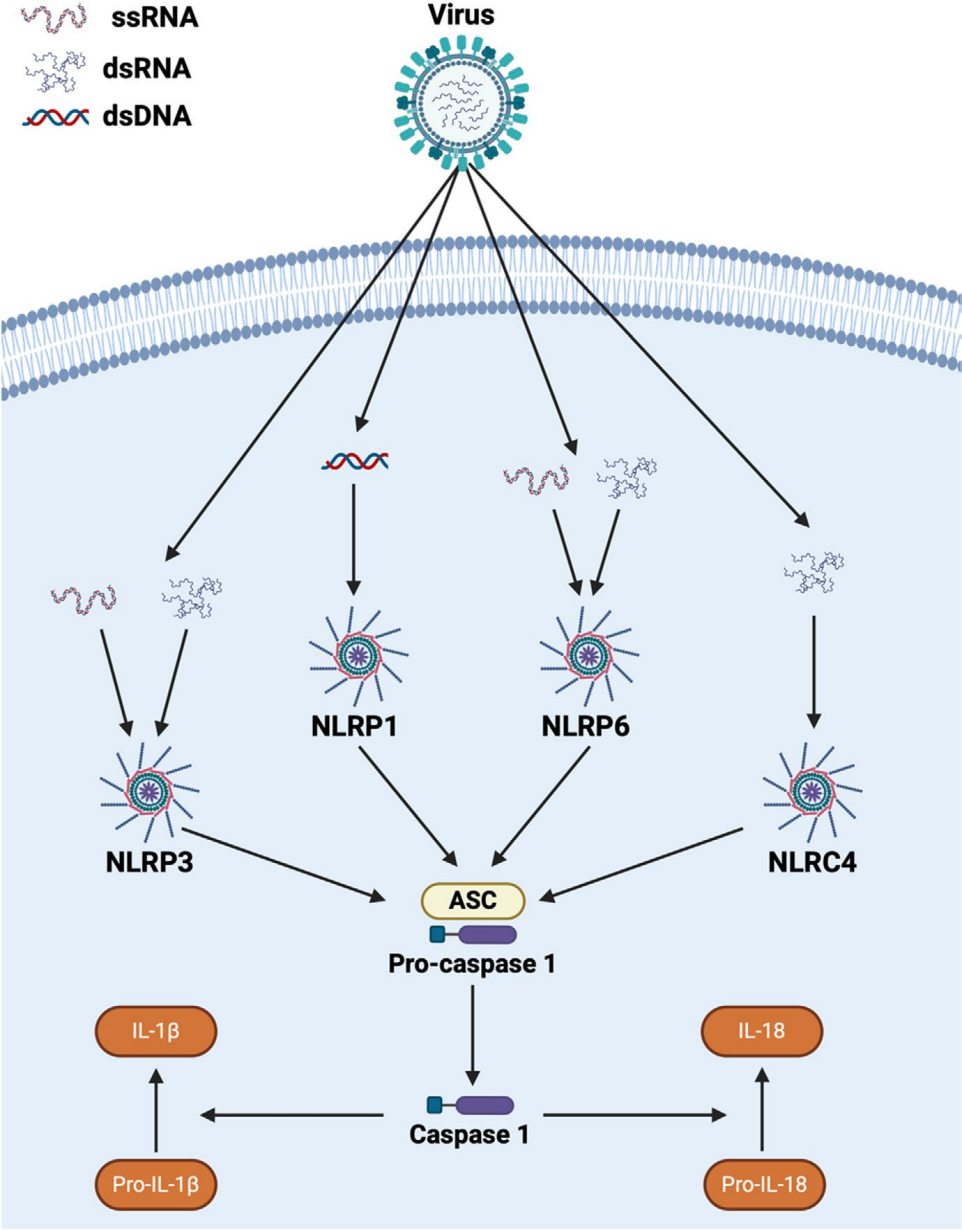
Activation of NLR Inflammasomes. Following viral entry, pathogen‐associated molecular patterns stimulate the formation of inflammasomes that subsequently promote the production of IL‐1β and IL‐18. NLRP1 is stimulated by dsDNA viruses. NLRP3 and NLRP6 can be stimulated by both ss and dsRNA viruses. NLRC4 is stimulated by dsRNA viruses.

NLRP3 is the best‐characterized NLR and senses a wide range of PAMPs and DAMPs. NLRP3 is composed of an LRR domain, a NACHT domain, and a PYD domain (Figure 1). NLRP3 is expressed in a variety of cells, including myeloid‐derived cells, epithelial cells, and neurons. NLRP3 has been shown to contribute to both canonical and noncanonical inflammasome formation. In the canonical signaling pathway, activation of other families of pattern recognition receptors (PRRs), such as the membrane‐bound Toll‐like receptors (TLRs), increases gene transcription and the expression of pro‐IL‐1β, pro‐IL‐18, pro‐caspase‐1, and even NLRP3 through NF‐κB signaling (Figure 3). This is termed “Signal 1”. Sensing of the virus and/or DAMPs associated with virus infection in the cytosol, such as dysregulated reactive oxygen species (ROS), by NLRP3 results in inflammasome formation. This is termed “Signal 2” and results in the cleavage and maturation of IL‐1β and IL‐18. Noncanonical NLRP3 inflammasome activation is associated with maturation of caspases 4 and 5 in humans, or caspase 11 in mice, and is associated with pyroptosis. This also results in an efflux of K+ that can further drive inflammasome formation [10]. In the context of host‐virus immune responses, the activation of NLRP3 and downstream signaling appears to vary based on ligand, cell type, and virus‐specific factors that are not yet fully defined.

The activation and downstream signaling of NLRP3 is important for the host defense against many viral pathogens, especially the release of IL‐1β. After infection with influenza A, mice deficient in NLRP3 had decreased survival and inflammatory cytokine production, suggesting an immune response dependent on NLRP3 activation. Mice lacking NLRP3 inflammasome components were shown to experience less airway inflammation, viral clearance, and IL‐1β release [11, 12]. The inhibition of lysosomal acidification, more specifically cathepsin B, was revealed to impair IL‐1β release, suggesting another potential mechanism for NLRP3‐mediated viral protection [11, 13]. Furthermore, lysosomal products were found to promote ROS generation in both human airway epithelial cells and murine in vivo models following influenza A infection [14]. THP‐1 cells in the presence of ROS inhibitors revealed abolished IL‐1β secretion following infection with A/Victoria/3/75 (influenza A) [11]. However, the exact mechanism of ROS inhibition of IL‐1β remains elusive. Stimulation with poly (I:C) and single‐stranded GU‐rich RNA (ssRNA40) was also found to stimulate NLRP3‐dependent signaling. Together, these data indicate that NLRP3 is activated through DAMP signaling and ssRNA following influenza A virus exposure [11].

While ROS signaling is clearly important for NLRP3 sensing and activation following influenza A virus exposure, it is established that ion channels play an important role in NLRP3 activation. For example, following exposure to Severe Acute Respiratory Coronavirus (SARS‐CoV), NLRP3 appears to be a significant mediator of the host innate immune response against the virus. The product of the viral transcription unit ORF3a has the capacity to induce gene transcription and maturation of IL‐1β through NLRP3 inflammasome formation [15]. The increase in gene transcription through Signal 1 is further associated with NF‐κB activation via TRAF3 ubiquitination of p105 in HEK293 cells [15]. Additional studies have further revealed that the SARS‐CoV‐1 envelope protein also activates the NLRP3 inflammasome through the formation of Ca+ ion channels [15, 16]. In HeLa cells deprived of ion channel activity, NLRP3 failed to localize in the perinuclear space, which is a hallmark event in NLRP3 inflammasome formation, and instead it maintained its diffuse localization, distributed throughout the cytosol [16]. ORF3a has also been shown to stimulate K+ channels and K+ efflux in an attempt to increase viral replication [17]. However, K+ efflux results in mitochondrial damage and increased ROS, which are also robust activation signals for the NLRP3 inflammasome [16, 18].

Beyond respiratory viruses, NLRP3 has also been implicated in sensing Myxoma virus (MYXV). Unlike the influenza A virus and SARS‐CoV, MYXV is a dsDNA virus and is a common model of viral immunomodulation due to a group of MYXV proteins that are able to recognize human homologous targets. MYXV has a high morbidity rate in European rabbits and is often studied in human cells but does not cause disease in humans [19]. In human monocyte cell lines (THP‐1), the maturation of pro‐IL‐1β was decreased at early time points following infection with MYXV however, there was no cleavage of caspase‐1 at these time points [20]. Rather, NLRP3 inflammasome activation appears to occur in response to lysosomal damage and cytoplasmic cathepsin B during MYXV infection [20]. THP‐1 cells expressing a MYXV construct devoid of the MO13 protein (vMyxMO13‐KO) had a significant decrease in IL‐1β secretion when treated with a cathepsin B inhibitor; however, when cells were not treated with a cathepsin B inhibitor, normal IL‐1β and NLRP3 inflammasome function was returned [21]. MO13 is a viral protein that contains a pyrin domain that has the ability to interact with ASC and inhibit the inflammasome complex. Furthermore, when ROS production was inhibited through NADPH oxidase inhibition in vMyxMO13‐KO THP‐1 cells, IL‐1β release was sequestered [21]. Thus, the virus encodes countermeasures to subvert inflammasome function through the MO13 viral protein, in this case through mimicking NLRs. However, when the virus is lacking the MO13 protein, cathepsin B and ROS production, NLRP3 activity is restored.

Viral countermeasures are critical considerations when evaluating NLRP3 inflammasome formation. In addition to MYXV, HSV‐1 also has the ability to subvert inflammasome signaling. HSV‐1 integrates into the host genome and establishes latent infections. In early stages of HSV‐1 infection, mature IL‐1β is commonly observed and appears to be associated, in part, with NLRP3 inflammasome activation. In studies using foreskin fibroblasts (HFF cells), NLRP3 colocalizes with ASC specks at the early HSV‐1 infection timepoints [22]. However, this co‐localization is transient in nature and not observed at later timepoints following HSV‐1 infection [22]. Mechanistically, it was hypothesized that HSV‐1 may suppress NLRP3 inflammasome signaling through the formation of actin “traps” to inhibit caspase‐1, similar to observations in previous studies [23]. HSV‐1 has the ability to promote changes in the cytoskeleton of infected cells, creating small actin bundle inflammasome components that must escape to form the inflammasome [23]. Actin clusters were observed in HFF cells infected with HSV‐1 by 4 h post‐infection, where caspase‐1 colocalizes and terminates NLRP3 inflammasome formation [23].

While NLRP3 inflammasome activation is critical to eradicating specific viruses from the host, in some cases this can actually augment disease pathobiology. For example, NLRP3 inflammasome activation is associated with the severe fever that occurs during thrombocytopenia syndrome (SFTS) associated with the SFTS virus (SFTSV) [24]. SFTSV is a tickborne disease with a relatively high mortality rate nearing 15% due to its ability to infect cells in the central nervous system that results in encephalitis or encephalopathy [25, 26]. While the mechanism is somewhat undefined, in studies using human microglial cells (MHC3), infection with SFTSV induced caspase‐1‐dependent cell death [24]. Studies using cell lines deficient in NLRP1, NLRP3, AIM2, and NLRC4 revealed roles for both NLRP1 and NLRP3 [24]. Here, IL‐1β and pyroptosis were reduced in cells lacking NLRP3. NLRP3 inhibitors significantly reduced viral replication in HMC3 cells compared to untreated controls [24]. Confocal immunofluorescence, coimmunoprecipitation, and western blot all confirmed that the non‐structural (NS) viral protein colocalized with NLRP3 when overexpressed and following SFTSV infection [24]. To further validate this interaction, the N‐terminal of the NS protein was tagged with HA (NSs1‐66) [24]. This tagged protein was shown to colocalize with NLRP3 and was found to be necessary for caspase‐1 cleavage [24].

### 2.2 NLRP1

Similar to NLRP3, NLRP1 also contains a PYD domain, the NACHT domain, and the LRR (Figure 1) [27]. However, it also contains a function‐to‐find domain (FIIND) and a CARD domain like CARD8, which aid in the enhancement of binding to caspase‐1 (Figure 1) [27]. While NLRP1 was one of the first NLR inflammasomes described, its biology is still relatively undercharacterized. This is in large part due to discrepancies between murine and human NLRP1 structure and, importantly, lineage‐specific duplications of *Nlrp1* with several paralogs located in tandem to one another in mice, creating variations in the *Nlrp1* gene locus that vary between mouse strains [28, 29]. Unlike humans, murine NLRP1 has three paralogs and lacks the N‐terminal PYD domain, suggesting an ASC‐independent mechanism associated with inflammasome formation in mice [30, 31]. In human cells, there is conflicting evidence related to NLRP1 inflammasome activation and its independence from ASC binding to the PYD domain [27, 32].

NLRP1 has a significant role in mediating the host immune response following virus exposure (Figure 3). Following exposure to dsRNA analog polyI:C, NLRP1 inflammasome formation was initiated in immortalized human keratinocytes. However, in HEK^NLRP1+ASC^ cell lines, NLRP1 did not form an inflammasome following challenges with polyI:C or a DNA analog polydA:dT, suggesting other mechanisms are necessary in these cells [33]. In human keratinocytes that express NLRP1, inflammasome formation was observed in response to the dsRNA viruses Semliki Forest virus (SFV) and Sindbis virus [33]. However, the NLRP1 inflammasome was not observed when challenged with vesicular stomatitis virus (VSV), which is a ssRNA virus [33]. In mechanistic studies, p38‐dependent ribotoxic stress was evaluated as a potential DAMP driving NLRP1 inflammasome formation. To evaluate this mechanism, immortalized human keratinocytes were challenged with dsRNA alphaviruses and treated with a p38 inhibitor [33]. NLRP1 was found to be directly activated by dsRNA, and ribotoxic stress appears to augment inflammasome formation [33]. Here, a linker region of the human NLRP1 protein, serine 107, was shown to be necessary for p38 phosphorylation [33]. This phosphorylation permits the ubiquitination of the PYD domain and subsequently initiates NLRP1 inflammasome formation [33]. This mechanism was not observed in HEK cells under the same conditions, revealing cell type‐specific mechanisms that underlie inflammasome formation [33].

Beyond just dsRNA, nucleic acid length appears to be a significant contributor to NLRP1 inflammasome formation following virus exposure. In human keratinocytes and bronchial epithelial cells, NLRP1 inflammasome formation was facilitated in response to long dsRNA viruses (> 500 bp) and through ATPase activity [34]. Immunoprecipitation confirmed viral dsRNA binding to human NLRP1 following SFV and polyI:C challenge [34]. Binding occurs within the LRR domain [34]. In the presence of long dsRNA and cytosolic viral dsRNA, the NACHT domain increases ATP hydrolysis activity, which facilitates ATP binding to the NACHT domain that drives the conformational change resulting in inflammasome formation [34, 35].

Consistent with these findings, the NLRP1 inflammasome is a key driver of the innate immune response following SARS‐CoV‐2 exposure. In A549 cells, which are human alveolar epithelial cells that were modified to express the human ACE2 receptor required for SARS‐CoV‐2 virus entry, the NLRP1 inflammasome was required for effective viral clearance [36]. Cells treated with remdesivir and PF‐00835231, which are both inhibitors of viral replication through interactions with the 2CL NSP5 protease, failed to form an inflammasome or ASC speck during influenza infection [36]. Similarly, inflammasomes and ASC specks failed to form when viral replication was inhibited through the 3CL proteases of MERS‐CoV, SARS‐CoV‐1, and SARS‐CoV‐2 [37]. The 3CL proteases are highly conserved across β‐coronaviruses [37]. Mechanistically, these proteases cleave NLRP1 in the NACHT domain at the NLRP1^Q333^ site [36]. However, this cleavage appears to be species‐ specific as this cleavage is not observed in murine NLRP1 [36]. This mechanism also extends beyond the β‐coronaviruses, as similar findings have also been reported for picornaviruses where the 3C proteases cleave human NLRP1 at sites Q^130^‐G^131^ [36]. SARS‐CoV‐2 also appears to target NLRP1 to inhibit gasdermin‐D‐(GSDMD)‐mediated pyroptosis [38]. The viral NSP5 protein is capable of cleaving GSDMD at Q^193^, which is a site that is different from the sequence targeted by caspase‐1 [36]. This reflects the importance of NLRP1 inflammasome formation and induction in pyroptosis in both limiting viral titer and in the recruitment of immune cells [36].

Unlike the findings in HEK cell lines, in human myeloid cells and keratinocytes NLRP1 has been implicated in inflammasome formation following dsDNA and in response to polydA:dT [38]. The cleavage and processing of IL‐1β in these cells appears to be at least partially associated with the formation of an NLRP1 inflammasome and independent of the cGAS‐STING‐NLRP3 pathway. Cleaved IL‐1β and gasdermin‐D levels are significantly attenuated in NLRP1 knockout keratinocytes following challenge with polydA:dT and dsDNA, but not ssRNA produced by polydA:dT [39]. In the recognition of dsDNA in keratinocytes, it appears that the formation of the NLRP1 inflammasome at least partially utilizes the MAPKKK ZAKα and also p38, similar to the results discussed above for dsRNA recognition during influenza infection [38].

As introduced above for SARS‐CoV‐2, viruses have distinct countermeasures targeting NLRP1. F1L and N1L proteins of the vaccina virus (VACV) are homologs with similar folding structures to host BCL‐2 family proteins that bind to pro‐apoptotic BCL‐2 proteins [40]. Certain motifs of BCL‐2 proteins have the ability to bind to NLRP1 [39]. Residues 32–37 on the C‐terminal end of F1L interact with and coimmunoprecipitate with NLRP1 [41]. Confirming these data, ΔF1L mutant viruses are unable to suppress NLRP1 inflammasome formation in a variety of human cell types, including macrophages (THP‐1), primary cultured peripheral blood mononuclear cells, and HEK293T cells [41]. The mutant virus was also unable to modulate cell death compared to the wild‐type virus [41]. Mice infected with the mutant strain experienced a reduced viral burden and showed an enhanced, early immune response to the virus [41].

Kaposi's sarcoma‐associated herpesvirus (KSHV) also has countermeasures targeting the NLRP1 inflammasome‐mediated immune response. Although the KSHV protein, ORF63, is relatively uncharacterized, *in silico* analysis has shown it to be a NLRP1 homolog; specifically, ORF63 mimics the NLRP1 LRR region [42, 43]. THP‐1 cells expressing ORF63 were primed with LPS to upregulate IL‐1β and stimulated with muramylidipeptide, a component of bacterial cell walls known to stimulate downstream signaling of NLRP1, and had attenuated IL‐1β and IL‐18 production; however, when ORF63 was silenced, IL‐1β and IL‐18 production was restored [43]. ORF63 was also shown to colocalize with NLRP1 by interacting with its NBD, LRR, and FIIND domains. Gel filtration chromatography revealed NLRP1 was fragmented into smaller molecular weights associated with ORF63 [43]. Together these data suggest ORF63 has the ability to inhibit NLRP3 inflammasome formation by preventing oligomerization. Considering the number of viruses that have evolved countermeasures targeting NLRP1, it is clear that inflammasome activation must play a significant role in the host immune response.

### 2.3 NLRP6

NLRP6 inflammasome formation has been highly studied in the gastrointestinal tract in the context of microbiome sensing [44]. However, NLRP6 is expressed in multiple tissues, including the lung and liver, where its biological functions are less defined [45, 46]. In recent mouse studies, NLRP6 has even been shown to act independently of inflammasome formation [47, 48]. NLPR6 forms the inflammasome complex through PYD‐PYD interactions with the ASC adaptor protein, which contains Arg39 and Trp50 residues in mice or R29/W50 residues in the human ortholog. These residues are necessary for inflammasome formation [47, 48]. The non‐inflammasome‐forming regulatory actions of NLRP6 appear to impact the interferon pathway and are independent of the R39/W50 residues [47]. Complementing these studies, *Nlrp6* silencing has shown that there is a decrease in RIG‐I and MAVS signaling resulting in altered mitophagy and mitochondrial dysfunction, which could also underlie some of the observations associated with altered interferon signaling [49]. In addition to interferon, NLRP6 also appears to negatively regulate NF‐κB and MAPK signaling through mechanisms that are independent of inflammasome formation, albeit these observations have been limited to models of bacterial exposure (Figure 3). Many of these mechanisms also appear to be based on cellular context [50]. It is important to note that these inflammasome‐independent mechanisms also appear to be compensatory, occurring when the inflammasome pathway is inhibited [47]. Thus, when considering the function of NLRP6 in host‐virus interactions, it is important to consider both its inflammasome and non‐inflammasome‐associated biological functions.

NLRP6 has been previously shown to interact with dsRNA, which promotes inflammasome formation. The C‐terminal of the LRR domain has a higher binding affinity for dsRNA with increased affinity as the number of base pairs increase [47]. In the presence of polyI:C, NLRP6 has been observed to form droplets as it is undergoing liquid–liquid phase separation (LLPS) in HBL‐1 cells [48]. LLPS is a mechanism behind the creation of biomolecular condensates, which transforms proteins into liquid, membrane‐less bodies used to coordinate biological processes, including transcription and signal transduction [51]. An intrinsically disordered poly‐lysine sequence (K350‐354A) was identified in the NACHT domain of NLRP6 that was determined to be necessary for the protein to undergo LLPS, gasdermin D cleavage, and caspase‐1 cleavage consistent with inflammasome formation [48]. The ASC speck formation and interferon‐stimulated genes were significantly reduced in models using NLRP6^K350‐354A^ genetically modified mice following murine hepatitis virus (MHV) exposure [48]. In addition to dsRNA, NLRP6 has also been shown to form an inflammasome following exposure to the negative‐sense ssRNA virus, vesicular stomatitis virus (VSV) [52]. Inflammasome formation is more robust in this model, potentially due to VSV countermeasures that have the ability to inhibit interferon signaling [52]. Mechanistically, NLRP6 inflammasome formation to viruses like VSV is relatively less characterized compared to that for dsRNA.

NLRP6 inflammasome formation has also been shown to be enhanced through the assistance of an RNA binding partner [53]. The RNA helicase DHX15, which is widely expressed in immune cells and is postulated to function as an RNA splicing factor or a viral RNA sensor [53]. Following EMCV or murine norovirus‐1 (MNV) infection, DHX15 co‐immmunoprecipitated with NLRP6 and facilitated NLRP6 inflammasome formation [54]. In NLRP6 knockout mouse studies, animals lacking NLRP6 deteriorated more rapidly compared to the wild‐type counterparts following infection with MNV, and this was found to be independent of the gut microbiome composition [55]. Furthermore, human intestinal epithelial cells and HEK293T cells infected with MNV revealed DHX15 was bound to NLRP6 only in the context of inflammasome formation [54, 55]. Furthermore, this interaction was specific to NLRP6, as DHX15 was not observed to interact with NLRP3, which has a highly similar protein structure [54]. This study suggests that the NLRP6‐DHX15 protein complex was able to facilitate the NLRP6 inflammasome formation, which subsequently attenuated viral replication and enhanced clearance through MAVS/RIG‐I interactions [54]. This observation was cell type specific, as the results were independent of MDA5 in the epithelial cells, which were contrary to findings in myeloid cells [54]. The NLRP6‐DHX15 protein complex was further found to be essential for the release of IFN‐β, IFN‐λ3, IL‐6, and IL‐18 through MAVs in human intestinal epithelial cells upon stimulation with polyI:C and infection with various RNA viruses [55]. However, the same effects were not observed following stimulation with dG;dC [55]. In sum, the role of NLRP6 in host‐virus interactions is still relatively undercharacterized.

### 2.4 NLRP9

NLRP9 is one of the least characterized NLRs. NLRP9 is one of a group of 3 NLRs that have undergone lineage‐specific duplications in rodents. Humans have a single NLRP9, defined as hNLRP9, while mice have three different isoforms, defined as mNLRP9a, mNLRP9b, and mNLRP9c. Originally, NLRP4, NLRP5, NLRP8, NLRP14, and NLRP9 were grouped as a sub‐group of NLRs associated with reproduction. All of these NLRs, with the exception of NLRP14, are tandemly distributed along chromosome 19, suggesting that a series of tandem duplication events likely led to their emergence [56].

NLRP9b has been implicated in the host immune response following rotavirus infection (Figure 3). In mouse models based on weaning pups, pups with NLRP9b deficiency demonstrated increased susceptibility to the virus [57]. It appears that NLRP9 has the capacity to form an inflammasome under specific experimental and perhaps in situ conditions. After controlling for the gut microbiome composition, epithelial cell‐specific *Nlrp9b* ^−/−^ mice were more sensitive to the virus [57]. Further mechanistic studies using HEK293T cells demonstrated that NLRP9 had a higher binding affinity for short viral dsRNA compared to other better‐defined sensors, including NLRP6 and RIG‐I [57]. Disrupting DHX9, which is also known to bind short dsRNA, disrupted the RNA‐binding affinity of NLRP9, whereas disruption of either DHX15 or RIG‐I did not have similar effects [57]. Together, this study identifies a novel NLRP9‐DHX9 protein interaction that senses short dsRNA to initiate inflammasome formation.

## 3 Regulatory NLRS

In general, inflammasome‐forming NLRs are the best‐characterized members of this pattern recognition receptor family. However, it is critical to realize that other functional sub‐groups of NLRs also exist that do not form inflammasomes. Regulatory NLRs have been defined that either positively or negatively regulate inflammation (Figure 2) [4]. Members of this sub‐group typically modify the function or signaling associated with other NLRs or pattern recognition receptors, such as RIG‐I‐like helicase receptors or TLRs. Functionally, all of the regulatory NLRs characterized to date still function through the formation of multi‐protein complexes, serving as scaffolds when activated, that are distinct from the inflammasome complex. These multi‐protein complexes are highly variable in protein composition and function. However, all have been implicated in regulating NF‐κB signaling, either positively or negatively, through distinct NLR‐specific mechanisms that are generally less defined compared to other families of pattern recognition receptors. Likewise, inflammation regulation is not limited to NF‐κB signaling, as many of the regulatory NLRs also modulate additional inflammatory signaling pathways, interferon signaling, autophagy, and cell death. Inflammasome signaling can be indirectly impacted through the function of these NLRs, for example, through the regulation of NF‐κB signaling that serves as the so‐called “Signal 1” that is associated with the gene transcription to generate the cell stores of pro‐IL‐1β, pro‐IL‐18, and pro‐caspase‐1. Regulatory NLRs are either positive regulatory NLRs or negative regulatory NLRs based on their generally characterized effects on inflammation signaling.

### 3.1 NOD1 and NOD2

NOD1 and NOD2 are positive regulatory NLRs and are members of the NLRC subgroup due to their CARD domains (Figure 1). Both proteins contain an NBD and LRR domain, while NOD1 has a singular CARD domain and NOD2 contains two CARD domains [58]. These pattern recognition receptors are best characterized for their recognition of D‐glutamyl‐meso‐diaminopimelic acid, peptidoglycans, and muramyl dipeptide, all found in Gram‐positive and Gram‐negative bacteria [59, 60, 61]. Once activated, the CARD domain typically interacts with the CARD domain of RIPK, ultimately activating the IFN, NF‐κB, and MAPK pathways [62]. However, while bacterial recognition is well documented, the exact mechanism/s of NOD1 and NOD2 activation and sensing remain relatively undefined, and their roles outside of host‐bacteria interactions are understudied.

**FIGURE 4:**
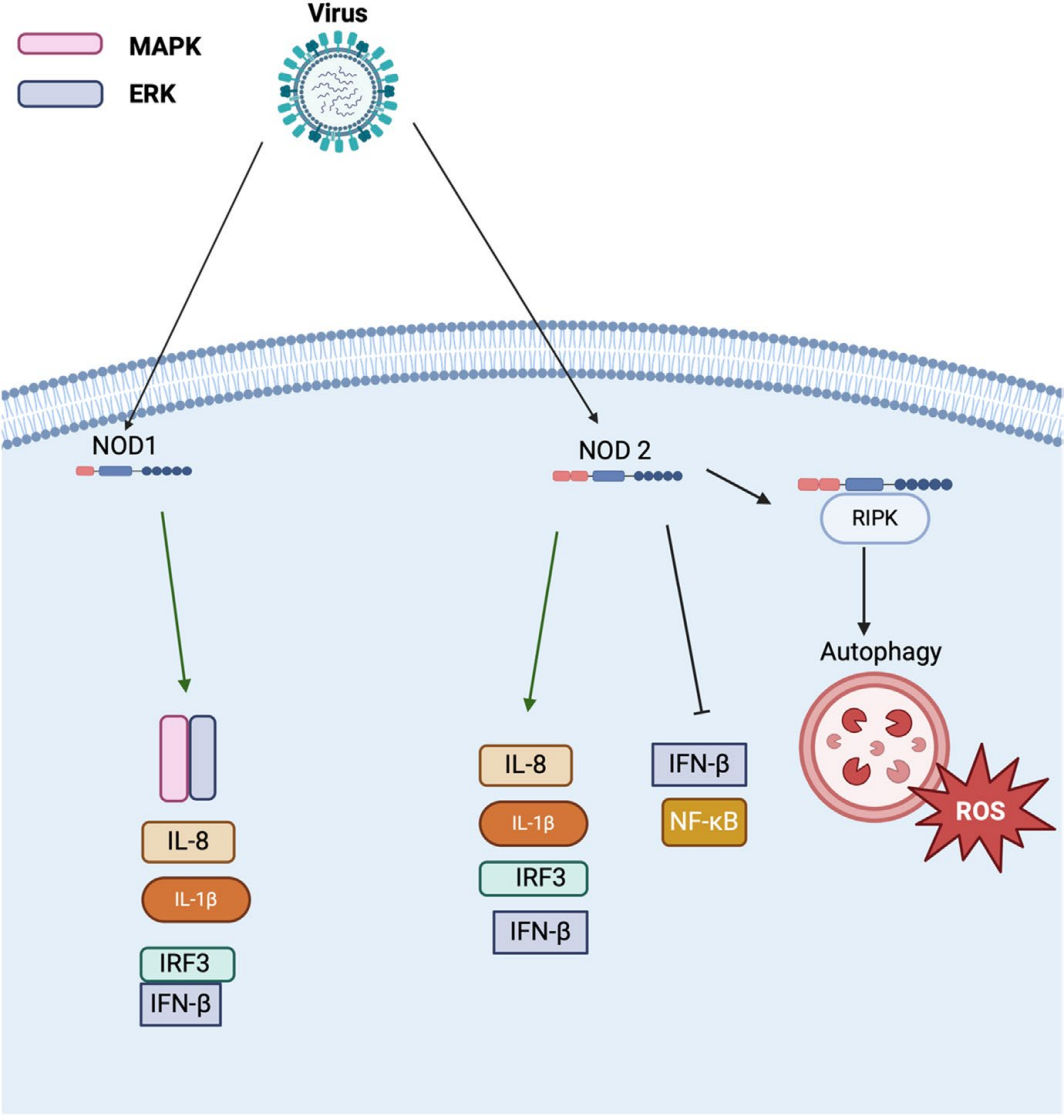
Regulatory Actions of Positive Regulatory NLRs. NOD 1 and NOD 2 are stimulated by viruses subsequently promote IL‐8, IFN‐ β and IL‐1β production, and IRF3 signaling. Furthermore, NOD1 has the ability to promote the MAPK/ERK complex. While NOD2 can promote IFN‐ β production, in the context of some viral conditions, it will inhibit IFN‐ β production and NF‐kB signaling. During influenza A infection, NOD2 forms a complex with RIPK to promote ROS production and autophagy.

In addition to bacteria‐associated PAMPs, both NOD1 and NOD2 appear to impact the host immune response to viruses in both model organisms and in human cells (Figure 4). In zebrafish models, infection with spring viremia or carp virus (SVCV) showed that larvae deficient in NOD1 demonstrated a significant impairment in their antiviral immune response [63]. This included a decrease in the expression of IRF3, IL‐1β, and IL‐8, with an increase in viral genes. Immunoprecipitation revealed a NOD1‐SCVC interaction, which was associated with the CARD domain of NOD1 binding to SVCV [63]. This is consistent with studies that demonstrated, in the absence of MDA5a, NOD1 has the ability to directly bind to polyI:C [63]. However, during infection with certain strains of SVCV, NOD1 appears to play a dichotomous role by augmenting the binding between dsRNA and MDA5a [63].

In humans, NOD1 regulates the host immune response following hepatitis C virus (HCV) infection. HCV is a dsDNA virus. In human hepatocytes, NOD1 expression significantly increases following infection; however, NOD2 levels are not significantly impacted [64]. Downstream, this corresponds to increases in MAPK/ERK and the production of several pro‐inflammatory cytokines associated with NOD1 [64]. Challenges with the NS5B protein of HCV in HEPaRG cells, which are human hepatocyte progenitors, also resulted in increased NOD1 expression [64]. Similar to the zebrafish studies detailed above, NOD1 was also found to bind to polyI:C in the HepaRG cells independent of RIG‐I/MAVS interactions, and inflammation, including IFN‐β release, appears to be dependent on NOD1 [64]. Further interactions have also been suggested to occur between NOD1 and TRAF3 that can also impact IFN production, albeit the mechanisms associated with and impacted by these findings and those discussed above are currently undefined. NOD2 regulates the host innate immune response following infection with foot‐and‐mouth disease virus (FMDV). Porcine kidney epithelial cells lacking NOD2 demonstrated attenuated IFN‐β and NF‐κB signaling compared to wild‐type cells [65]. This appears to be associated with an impairment in the phosphorylation of RIP2. Further mechanistic studies revealed that the viral proteins 2b, 2c, and 3c^pro^ were capable of downregulating NOD2 during infection [65]. Furthermore, NOD2 is found to be important for the immune response to influenza A. *Nod2* ^*−/−*^ murine models infected with influenza A/Puerto Rico/8/34 (PR8) had increased disease burden and decreased survival compared to wild‐type mice [66]. Previous studies have shown NOD2 to regulate type I interferon signaling following IAV infection [67, 68]. Furthermore, interactions between RIPK2 and NOD2 were stimulated to promote autophagy, only in the presence of IAV viral RNA [66]. Autophagy induced during RIPK2 and NOD2 interactions leads to mitochondrial damage and release of ROS, promoting the activation of the NLRP3 inflammasome to protect against IAV infection [66]. However, beyond these limited studies, the roles and mechanisms associated with NOD1 and NOD2 during host‐virus interactions remain relatively understudied.

## 4 Negative Regulatory NLRS

### 4.1 NLRP12

**FIGURE 5:**
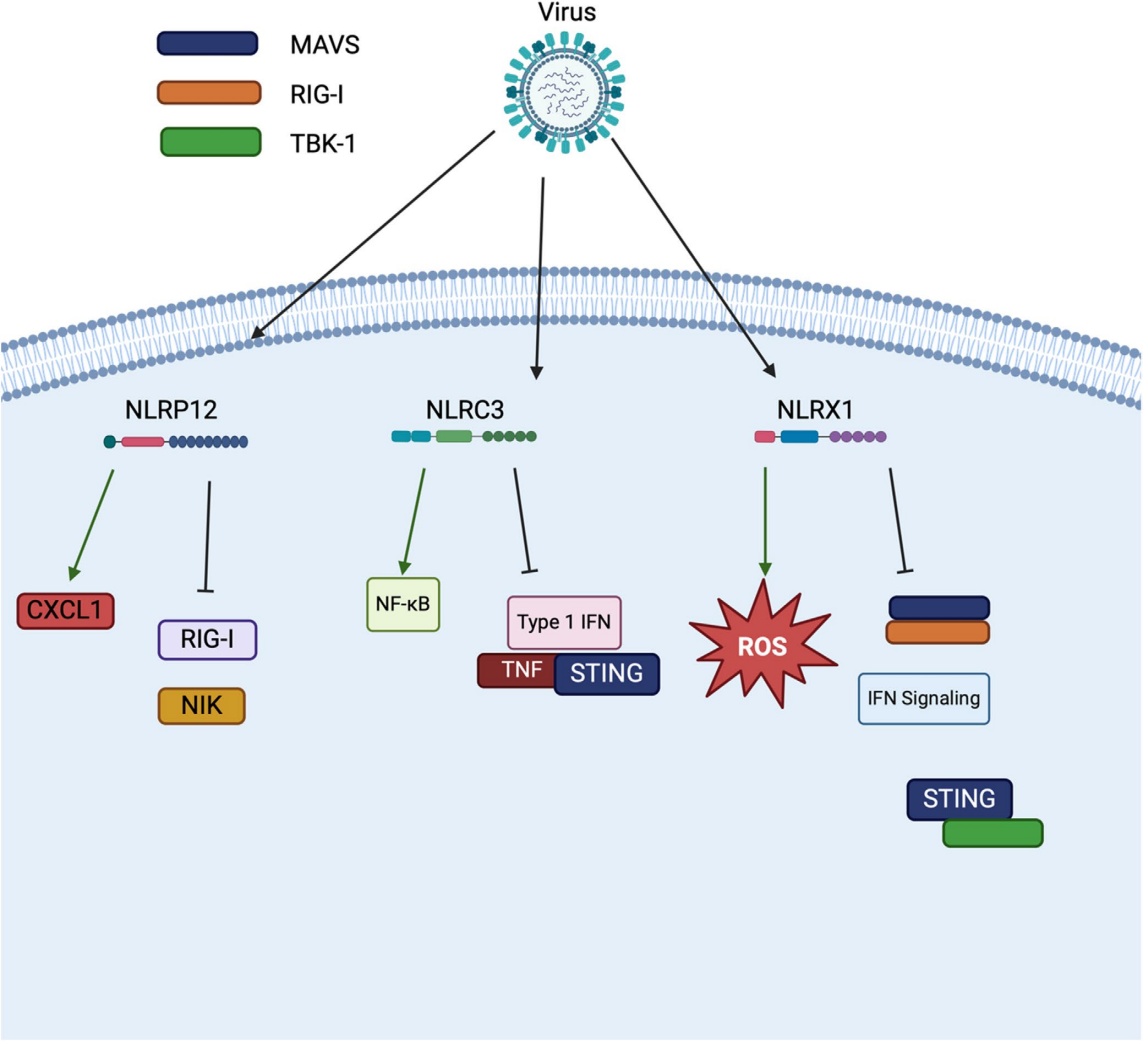
Downstream Actions of Regulatory NLRs. Following viral infection regulatory NLRs regulate downstream immune signaling. NLRP1 is shown to promote CXCL1 production while inhibiting the actions of RIG‐I and NIK signaling. NLRC3 can promote NF‐kB, but in certain cell specific contexts inhibit TNF, type 1 IFN, and STING signaling. NLRX1 is shown to inhibit singaling through IFN, and disrupting the MAVS/RIG‐I complex, and STING/TBK‐1. NLRX1 is shown to promote production of ROS.

Under certain experimental conditions in response to PAMPS and DAMPS, NLRP12 can form an inflammasome through complexing with ASC; however, this is rare in the context of viral infections [69]. The majority of data indicates that NLRP12 functions to negatively regulate inflammatory signaling under most physiologically relevant scenarios [69]. This attenuation is associated with negative regulation of canonical and noncanonical NF‐κB signaling and MAPK signaling [70, 71]. NLRP12 consists of a PYD domain, a central NACHT domain, and C‐terminal leucine‐rich repeats (Figure 1) [69]. NLRP12 does not appear to directly interact with PAMPs. Rather, it appears that NLRP12 binds to cytosolic host proteins, such as heat shock factors, the IKK complex, and interleukin‐1 receptor‐associated kinase 1 (IRAK1) [71, 72]. Additionally, NLRP12 has been suggested to interact with TRAF3 and the NF‐κB‐inducing kinase (NIK), which induces ubiquitination and the proteasomal degradation of NIK to inhibit the noncanonical NF‐κB pathway (Figure 5) [71].

NLRP12 has been implicated in host anti‐viral immune regulation of inflammation initiated from other pattern recognition receptors. Following Dengue virus (DENV) in macrophages, NLRP12 appeared to regulate the immune response [73]. In studies using human macrophages polarized to an M1 state and infected with DENV, levels of NLRP12 mRNA transcripts were reduced while protein levels of PRDM1, which is an inhibitor of NLRP12, were increased [71]. In similar studies, when NLRP12 was silenced with small interfering RNA, DENV burden increased, whereas, when NLRP12 was present, virus burden decreased [73].

NLRP12 is a significant regulator of immune function in mucosal tissues, with many characterization studies focused on the lungs and gut. In the respiratory tract, NLRP12 attenuates the production of the chemokine CXCL1, which controls neutrophil chemotaxis and a variety of other generally pro‐inflammatory functions [73, 74, 75]. Using *Nrlp12* ^*−/−*^ mice in influenza virus (IAV) infection models, animals lacking NLRP12 exhibited increased survival compared to the wild‐type counterparts [74]. The improved survival could be associated with an enhanced CD4+ and CD8+ T‐cell functionality and response; however, it should be noted that no significant differences in the magnitude of the T‐cell response to IAV infection were observed between *Nlrp12* ^*−/−*^ and wild‐type mice [74]. There was a significant decrease in CXCL1 in the *Nlrp12* ^*−/−*^ mice, but no other differences in chemokines or cytokines were statistically significant between genotypes. Together, these studies suggest that NLRP12 regulates CXCL1 in response to IAV, which likely impacts neutrophil recruitment, vascular permeability, and potentially T cell functionality (rather than numbers of T cells).

In addition to IAV, NLRP12 appears to also have a role in SARS‐CoV‐2 infection, albeit a direct function is yet to be defined. A group of non‐structural proteins from SARS‐CoV‐2 directly interact with NLRP12. Specifically, NLRP12 is cleaved by the 3C‐like protease (3CLpro), which is encoded within the nonstructural protein 5 (NSP5) [76]. Two binding and cleavage sites have been identified for NLRP12. One binding site was confirmed and identified as residue 938 on the C‐terminal of NLRP12, whereas the second cleavage site remains undefined [76]. This cleavage of NLRP12 results in the lost ability to negatively regulate NIK and may directly contribute to the cases of hyper‐inflammation observed in COVID‐19 patients [76]. The targeting of NLRP12 by the virus likely confers a selective advantage that is currently undefined.

NLRP12 regulation is not always protective against viral infection. For example, during vesicular stomatitis virus (VSV) infection, NLRP12 prevents the downstream signaling of RIG‐I, an important viral mediator. Murine dendritic cells void of NLRP12 show heightened immune signaling of downstream RIG‐I effectors, including TBK1 and IRF3, following VSV stimulation [77]. TRIM25, an adaptor protein needed for RIG‐I activation, was found to colocalize with NLR12 decreasing the association between TRIM25 and RIG‐I. VSV‐infected murine models deficient in NLRP12, revealed a more robust IFN response and viral burden [77]. RIG‐I expression was also increased in NLRP12 void mice. Murine models of myeloid conditional Nlrp12‐deficient mice showed a similar phenotype [77]. Together this suggests NLRP12 inhibits the immune response to VSV by competitively binding TRIM25 and blocking the association of TRIM25 and RIG‐I. It should be noted that the timing of these processes is still unclear. These could reflect dysregulated temporal inhibition of anti‐viral signaling, which would be important later in the host immune response to quell these signaling pathways and facilitate inflammation resolution.

### 4.2 NLRC3

NLRC3 negatively regulates inflammation through interactions with STING and components of the NF‐κB signaling pathway, resulting in attenuated IFN and cytokine production [78]. NLRC3 senses viral DNA and has shown an affinity for binding dsDNA in the C‐terminus of the receptor [78]. This binding results in an increase in the ATP hydrolysis activity of the NACHT domain, resulting in a conformational change that disassociates NLRC3 from sequestered STING, permitting STING to recruit TANK‐binding kinase 1 (TBK1) [78]. Activated TBK1 phosphorylates STING, resulting in phosphorylation of the transcription factor interferon regulatory factor 3 (IRF3), allowing IRF3 to translocate to the nucleus to promote type I interferon production [78]. Following activation by STING, TBK1 can also activate the IKK complex, resulting in downstream activation of NF‐κB, which acts synergistically alongside IRF3 to increase proinflammatory cytokines [79]. In addition to complexing with STING, NLRC3 also interacts with TRAF6 in a complex coined the “TRAFasome” and prevents TRAF6 from ubiquitinating its target proteins [80]. This includes components of the canonical NF‐κB signaling pathway, which further attenuates inflammatory signaling (Figure 5) [80].

NLRC3 senses herpes simplex virus 1 (HSV‐1) dsDNA, which binds to the LRR domain of the protein [81]. This binding between NLRC3 and HSV‐1 dsDNA releases sequestered STING and TBK1, allowing them to interact and attenuate the downstream type I IFN response [81]. Indeed, IFN‐I protein levels are increased in immune cells derived from *Nlrc3* ^*−/−*^ mice [81]. These findings extended to cells beyond the immune system. Specifically, mouse embryonic fibroblasts from *Nlrc3* ^*−/−*^ mice also demonstrated an increase in type I IFN production following HSV‐1 infection compared to wild‐type counterparts [80]. In addition to IFN, HSV‐1 infection also resulted in increased TNF levels in immune cells from *Nlrc3* ^*−/−*^ mice [81]. These findings suggest NLRC3 inhibition of type 1 IFN signaling.

In addition to HSV‐1, NLRC3 also modulates inflammation following human papillomavirus (HPV) infection [82]. Release of HPV dsDNA into the cytosol is facilitated by endocytosis, followed by viral‐endosome membrane fusion. It is at this point that the dsDNA may interact with the C‐terminus of NLRC3 [82]. The exact mechanism of this interaction is unclear and yet to be defined. However, it should be noted that STING signaling has been implicated in HPV and head and neck squamous cell carcinoma, and destabilization of this pathway may impact the resultant immune response [82]. Thus, NLRC3‐STING interactions are highly probable mechanisms associated with immune regulation following HPV exposure. NLRC3 can also recognize and bind saturated fatty acids, which can both promote increased expression of NLRC3 and the concomitant downregulation of STING signaling and type I IFN production [82]. HPV‐positive dysplastic cells have a higher lipid content compared to normal cells, which may be an additional mechanism driving NLRC3 activation [82].

The immune response to another dsDNA virus, Epstein–Barr virus (EBV), is known to be regulated by NLRC3. B lymphocytes expressing LMP1, critical for viral production, revealed a decrease in NLRC3 expression; however, when LMP1 is silenced, NLRC3 expression is restored [83, 84]. Similarly, B cells with latent EBV also had decreased NLRC3 transcription [85]. LMP1 is also reported as inducing NF‐kB in HEK293 cells [84]. Interestingly, cells devoid of NF‐kB and overexpressing LMP1 did not suppress NLRC3 expression, suggesting a potential mechanism [84]. Although NLRC3 expression is downregulated by LMP1‐induced NF‐kB activation, overexpression of NLRC3 attenuated this activity by increasing the degradation of LMP1 in HEK‐293T cells. Herpesviruses are known for their ability to stay dormant in a host cell by silencing the viral genome and avoiding the host immune response [85]. In *Nlrc3* ^*−/−*^ mice infected with MHV‐68, a murine gammaherpes virus, copy number of viral genomes and frequency of viral reoccurrence from latency were increased compared to wild‐type controls, suggesting NLRC3 may protect against viral latency [84].

NLRC3 has a greater affinity for binding dsDNA and has been better defined in this context. However, NLRC3 has also been shown to modulate the host immune response to RNA viruses. Specifically, Hantaan viral infection (HTNV) resulted in a higher viral burden in *Nlrc3* ^*−/−*^ mice compared to wild‐type animals [86]. Conversely, TNF levels and other NF‐κB‐associated cytokines were significantly increased in the absence of NLRC3. These data suggest that despite the increased pro‐inflammatory signaling associated with increased NF‐κB, HTNV was able to significantly proliferate [86]. This likely reflects a currently unknown mechanism underlying NLRC3 in HTNV infection that perhaps even extends to additional RNA viruses.

### 4.3 NLRX1

Among the negative regulatory NLRs, NLRX1 is the most studied and appears to have the most complex functions in the regulation of viral immunity. NLRX1 is widely expressed in most cell types and tissues. Like the other NLR family members, the protein consists of a central NACHT domain, and sensing occurs along the C‐terminus through the LRR domains (Figure 1). However, the N‐terminus of NLRX1 is relatively undefined beyond a mitochondrial targeting sequence [87]. NLRX1 plays a critical role in regulating mitochondrial function, metabolism, ROS signaling, autophagy, cell death, cell homeostasis, and immune signaling following pathogen exposure and in cancer model [87]. Importantly, the function and activation of NLRX1 appear to be cell type and pathogen specific [88, 89]. NLRX1 directly senses viral RNA and is capable of binding either single‐stranded RNA (ssRNA) or double‐stranded RNA (dsRNA) [90]. However, NLRX1 does not appear to directly bind DNA. Once activated, NLRX1 acts as a scaffold and regulates downstream signaling complexes to facilitate its various biological functions (Figure 5) [90].

NLRX1 was originally characterized for its role in attenuating overzealous inflammation following influenza A virus (IAV) infection [91]. IAV is a segmented ssRNA virus and distributes its nucleic acid into the cytosol via viral‐endosome membrane fusion. IAV ssRNA is sensed by the RIG‐I pattern recognition receptor, which interacts with the mitochondrial antiviral signaling (MAVS) protein. Binding of IAV viral RNA by RIG‐I induces a conformation change in the tertiary structure of RIG‐I, which exposes its CARD domain and facilitates binding to MAVS through CARD‐CARD interactions. MAVS is localized to the mitochondria and forms filaments along the outer membrane. The RIG‐I/MAVS complex is termed the “signalosome” [92]. Signalosome formation results in type I interferon signaling and NF‐κB signaling. Mechanistically, RIG‐I/MAVS signaling results in the recruitment and activation of the tumor necrosis factor receptor‐associated factor (TRAF) family of ubiquitinases, specifically TRAF6. Under normal cellular conditions where NLRX1 is endogenously expressed, NLRX1 outcompetes RIG‐I and inhibits the interaction with MAVS [93]. Conversely, in the absence of NLRX1, there are increased RIG‐I and MAVS interactions, resulting in increased inflammatory mediator output [91]. Using *Nlrx1* ^*−/−*^ mice, IAV infection resulted in a significant increase in IFN signaling and the production of IFN‐β compared to wild‐type mice, leaving *Nlrx1* ^*−/−*^ more susceptible to infection by increasing airway epithelial cell denuding, obscured small airways, and IL‐6 production [91]. NLRX1 appears to specifically inhibit RIG‐I signaling, as studies using NLRX1‐deficient cells and encephalomyocarditis virus (ECMV), which is recognized through the related RIG‐I‐like helicase receptor MDA5, did not result in significant differences in IFN‐β output [91].

In addition to influenza, NLRX1 has also been evaluated following hepatitis B virus (HBV) exposure. HBV is a DNA virus. However, upon infection of the host cells, HBV releases a single‐stranded RNA pre‐genome into the cytosol, which is targeted by a viral reverse transcriptase to facilitate the virus replication cycle [94]. This RNA pre‐genome can be sensed by NLRX1 [94]. In HepG2‐NTCP cells infected with HBV, the RIG‐I/MAVS signaling pathway was activated, which suggests a possible mechanism for NLRX1 function [94]. When HBV infects cells, the mitochondrial membrane potential is significantly altered, and the production of reactive oxygen species is increased [95]. NLRX1 is directly engaged with the outer mitochondrial membrane through its N‐terminus and can regulate ROS production. In HeLa cell lines, overexpression of NLRX1 results in increased ROS production [96]. ROS production can effectively neutralize viral replication, albeit it also causes significant collateral damage to the host cell [97]. NLRX1 can also interact with the mitochondrial electron transport chain (ETC) through the ubiquinol‐cytochrome C reductase core protein 2 (UQCR2) located within the respiratory chain complex III of the mitochondrial ETC. [96] Through this interaction, NLRX1 can cause the transport of electrons along the mitochondrial ETC to leak, driving the generation of ROS, such as superoxides, hydrogen peroxide, and other free radicals [97]. Thus, it is also possible that NLRX1 modulates the host immune response to HBV, as well as other viruses, through the regulation of ROS production. Interestingly, NLRX1's modulation of ROS seems to be independent of the C‐terminus of the receptor being bound by a viral PAMP [96].

Unlike the protective role of NLRX1 discussed above, NLRX1 is found to be detrimental to the host response to HIV‐1. HIV‐1 is a dsDNA virus. When NLRX1 is silenced in THP‐1, monocyte‐derived macrophages and dendritic cells, HIV‐1 has attenuated viral replication, suggesting NLRX1 may be responsible for enabling replication [97]. Low *Nlrx1* expression is associated with a more robust and productive immune response, specifically the type 1 IFN response, following HIV‐1 infection [97]. HIV‐1 DNA is recognized by cGAS, initiating the production of cGAMP used to activate STING [97]. *Nlrx1* ^*−/−*^ mouse embryonic fibroblasts showed increased STING activation and interferon‐stimulated proteins [97]. Furthermore, in HEK293T cells, NLRX1 colocalized with STING in the mitochondrial matrix to inhibit the STING‐TBK1 interaction necessary for STING to recognize dsDNA [97]. In the absence of NLRX1, IFNβ1 and MX2 transcription is attenuated following HIV‐VSV infection [97]. However, in the absence of STING, there is no transcription of IFNB1 and MX2 [97]. Similarly, this phenomenon is reported in NLRX1 mice infected with HSV‐1, suggesting NLX1 may negatively regulate the host immune response to DNA Viruses [97].

## 5 Concluding Remarks

Significant progress has been made over the last decade in understanding the biological functions of the NLR family of pattern recognition receptors in host‐virus interactions. Members of all three NLR subgroups appear to participate at some level in regulating the host anti‐viral immune response. While several NLRs function through well‐characterized and/or well‐studied mechanisms, it is evident that we still have significant gaps in knowledge across all the members of this pattern recognition receptor family. This is especially true for the regulatory NLRs and the family members that appear to function to attenuate inflammation following virus infection. Future studies will hopefully address outstanding issues related to binding partners and multi‐protein complex formation, NLR‐virus interactions and specificity, signaling mechanisms, cell type‐specific functions, and temporal regulation. Better defining these mechanisms will significantly improve our overall understanding of host antiviral immune responses.

## Conflicts of Interest

The authors declare no conflicts of interest.

## Data Availability

The authors have nothing to report.
